# Influence of Factors of Cryopreservation and Hypothermic Storage on Survival and Functional Parameters of Multipotent Stromal Cells of Placental Origin

**DOI:** 10.1371/journal.pone.0139834

**Published:** 2015-10-02

**Authors:** Denys Pogozhykh, Volodymyr Prokopyuk, Olena Pogozhykh, Thomas Mueller, Olga Prokopyuk

**Affiliations:** 1 Institute for Problems of Cryobiology and Cryomedicine, National Academy of Sciences of Ukraine, Kharkiv, Ukraine; 2 Institute for Transfusion Medicine, Hannover Medical School, Hannover, Germany; Wake Forest Institute for Regenerative Medicine, UNITED STATES

## Abstract

Human placenta is a highly perspective source of multipotent stromal cells (MSCs) both for the purposes of patient specific auto-banking and allogeneic application in regenerative medicine. Implementation of new GMP standards into clinical practice enforces the search for relevant methods of cryopreservation and short-term hypothermic storage of placental MSCs. In this paper we analyze the effect of different temperature regimes and individual components of cryoprotective media on viability, metabolic and culture properties of placental MSCs. We demonstrate (I) the possibility of short-term hypothermic storage of these cells; (II) determine DMSO and propanediol as the most appropriate cryoprotective agents; (III) show the possibility of application of volume expanders (plasma substituting solutions based on dextran or polyvinylpyrrolidone); (IV) reveal the priority of ionic composition over the serum content in cryopreservation media; (V) determine a cooling rate of 1°C/min down to -40°C followed by immersion into liquid nitrogen as the optimal cryopreservation regime for this type of cells. This study demonstrates perspectives for creation of new defined cryopreservation methods towards GMP standards.

## Introduction

Rapid development of regenerative medicine and cell therapy stimulates high demand for multipotent stromal cells (MSCs), due to their proven efficiency in the treatment of numerous severe pathologies of the nervous, cardiovascular, reproductive, and endocrine systems; furthermore skin lesions and venous ulcers [1,2,3].

Although to date different tissues and organs serve as the source of MSCs [3,4], the most promising sources are various structural components of placenta including placental villi, fetal membranes and the umbilical cord, mainly because such sources allow extraction of the highest number of MSCs [2,5,6,7]. Moreover, due to the developmental origin of these tissues being partially fetal and partially maternal, it is possible to simultaneously receive two different patient-specific materials, one for mother and the other for the fetus [8]. Thus, the merit of the placenta as a source of MSCs is obvious due to the natural availability of large amounts of possibly autologous material without invasive surgery and comparatively simple procedure of cell isolation [2,6,9,10].

Necessity for storing placental biological objects originated from the early attempts in the beginning of 20th century until nowadays, where regenerative medicine and stem cell therapy changed the focus of cryopreservation on placental cell suspensions, cord blood, umbilical cord and fetal membranes [11,12,13]. Cryopreservation methods common for majority of cell suspensions are also currently applied for storage of the placental MSCs with varied outcome. However, they need to be improved according to specific requirements of particular biomaterials and modern standards in clinical practice [14,15].

Hypothermic storage of placental cells may be necessary for transporting of biological material over short distances between the laboratories and, possibly, in clinics. Unlike cryopreservation, hypothermic storage does not require specialized cryo equipment; therefore it simplifies short-term preservation of material for a period of a few days, which may allow transporting the samples to distant locations, or storing in a medical institution. Hypothermic conditions, relative to the body temperature of the human, can be provided by refrigeration equipment, from +4°C to +8°C, as well as in the conditions of subnormothermic (room) temperature from +18°C to +25°C.

Hypothermic storage technologies were developed primarily for organs or tissue fragments for transplantation purposes, where cryopreservation is complicated [16,17]. Usually, special solutions containing macromolecular colloids, carbohydrates, antioxidants and energy compounds are used for hypothermic storage to minimize the effect of ischemia-reperfusion injury [18,19]. Moreover, some researchers have demonstrated that subnormothermic conditions cause reduction of metabolic rate and allow short-term storage of cells and tissues more efficiently in comparison to normothermic conditions (+37°C) [20,21].

However, only the low temperature cryopreservation ensures possibility of long-term storage of MSCs of different origin; therefore constant improvement of low-temperature storage and transportation technology is the essential condition for clinical application of cellular material. On the one hand it should provide high viability of cryopreserved material and on the other—comply with GMP and GLP standards. Wide range of cryoprotective media with varied efficiency rates is offered today for cryopreservation of stem cells [22]. However, to avoid possible clinical complications and following legislative issues, the vast majority of such media is labelled as “for scientific purposes only”, “not for drug, human, or veterinary use”, and etc. Nevertheless, it is crucially important to utilize cryopreservation technology and materials that are approved for medical use, while serum of animals, many cell culture media and a number of cryoprotective agents (CPA) are not allowed for clinical application in most of the countries, as well as not all equipment is certified for such purposes [14,15].

Besides the components of cryoprotective media, the cooling temperature regime is one of the key parameters of low-temperature preservation and long-term storage [23]. Freezing with the rate of 1°C/min to -80°C and subsequent immersion into liquid nitrogen is the commonly accepted procedure for long-term preservation [24]. The efficiency of this method is connected with temperature conditions required for saturation of the sample with CPA, optimal crystal formation and prevention of recrystallization during thawing. Besides, such protocol meets necessity for practical simplification in the laboratory routine freezing procedure handling, like commercially available cryo-containers (e.g. Nalgene^®^ "Mr. Frosty" freezing container), which lower the temperature at such rate due to the slow cooling properties of isopropyl alcohol. This also permits elimination of necessity for programmable freezers. Further liquid nitrogen based low temperature storage at -196°C is to be preferred due to standardization and simplicity of the process. However, refrigeration equipment with the end temperature of −40°C −80°C became widely available in recent years not only in the research laboratories, but in clinics and blood donation centers as well. Moreover, in accordance with the recommendations of the World Health Organization, certain blood components should be stored at a temperature of less than -40°C (WHO Recommendations for the Production, Control and Regulation of Human Plasma for Fractionation. ECBS 2005. WHO/BS/05.2019). The legislation of Ukraine, for example, also determines the temperature of storage of certain blood components at -30°C—-80°C (Order of the Ministry of Health of Ukraine of 17.12.2013 № 1093). Thus, it is necessary to study cryosensitivity of placental MSCs and other therapeutically important stem cell types in this temperature range and analyze necessity and possibilities for modification of existing cryopreservation protocols.

Therefore, in this paper we aim to analyze the influence of various factors of cryopreservation and hypothermic storage on survival and functional parameters of multipotent stromal cells of placental origin.

## Materials and Methods

### Experimental design

In summary, placental amnion cells were isolated by a standard enzymatic method, characterized for MSC origin as described before [25], and followed by comprehensive comparative analysis of cells in regular culture conditions with the cells after hypothermic storage at +4°C, subnormothermic storage at +20°C, after cryopreservation with varied composition of cryoprotective medium, different temperature and cooling rate regimens and after repeated freezing.

### Cell derivation, isolation, and culture

#### Ethical and legislative statement

No animal experiments were performed in this study. Donation of the human placenta material after routine Caesarian section was performed in an anonymized manner and a written informed consent of the patients at the Department of Gynaecology and Obstetrics at Hannover Medical School, Germany (approved by Ethical Commission of Hannover Medical School, Ethic votum No.2396-2014) and in Kharkiv municipal maternity hospital No.1, Ukraine (approved by Bioethics Committee of the Institute for Problems of Cryobiology and Cryomedicine of the National Academy of Sciences of Ukraine, Ethic votum No.2-0306-2013).

#### Cell derivation and isolation

Primary culture of MSCs was obtained from placental amnion by enzymatic method [25]. Amnion membranes were washed with PBS with 10% Ciprofloxacin (Fresenius Kabi, Bad Homburg, Germany), then dissected into small pieces and incubated in presence of 0.25% trypsin for 1 hour at 37°C. Following trypsin digestion, samples were filtered through 100 μm cell strainer (BD Biosciences, Durhan, USA), the cell suspension was centrifuged for 5 min at 1200 rpm (Heraeus Multifuge 1S-R, Thermo Fisher Scientific GmbH, Dreieich, Germany), the cell pellet was resuspended in MSC growth medium and plated into 10 cm cell dishes (Cellstar, Greiner BioOne, Frickenhausen, Germany).

#### Cell culture

MSCs derived from placenta were cultured under sterile conditions in Dulbecco’s modified Eagle’s medium (DMEM, Biochrom AG, Berlin, Germany) containing 10% (v/v) fetal bovine serum (FBS, Lonza, Belgium), 1% antibiotic-antimycotic solution (BioWest, Nuaillé, France), and 1% ascorbic acid (Sigma-Aldrich, St. Louis, USA) in 10 cm tissue culture dishes (Cellstar, Greiner BioOne, Frickenhausen, Germany) in a humidified CO_2_ incubator (Thermo Fisher Scientific GmbH, Dreieich, Germany) at 5% CO_2_ and 37°C. The cells were harvested using 0.25% trypsin solution.

### Multipotent stromal cells characterization

#### Reverse transcription polymerase chain reaction (RT-PCR) for MSC markers

RNA extraction was performed with application of peqGOLD Total RNA Kit (Peqlab GmbH, Erlangen, Germany) according to manufacturer’s protocol. In brief, the cell pellet was lysed in 400 μl RNA lysis buffer and transferred to a DNA removing column to remove contaminant DNA. After centrifugation at 12,000 x g for 1 min, 400 μl of 70% ethanol was added to the flow through. The lysate was loaded onto a Perfect Bind RNA Column and centrifuged at 10,000 x g for 1 min to bind the RNA to the column,followed by a washing step with 500 μl RNA Wash Buffer I and two times washing with 600 μl RNA Wash Buffer II. The column was dried by centrifugation at 10,000 x g for 2 min and total RNA was eluted from the column by applying 50 μl sterile RNase-free water. The RNA concentration was measured with a NanoDrop photometer ND–1000 (Thermo Fisher Scientific GmbH, Dreieich, Germany). Extracted RNA was transcribed into complementary DNA (cDNA) using the High Capacity cDNA Reverse Transcription Kit (Life Technologies GmbH, Darmstadt, Germany). Adding Oligo (dT) primers (TIB Molbiol, Berlin, Germany) ensured that only the mRNA is transcribed. For analysis of mesenchymal markers a reverse transcription PCR reaction was set up in 30 μl volume per sample as follows: 24 μl ddH_2_O (double distilled water), 3 μl 10x PCR buffer (NEB, Frankfurt, Germany), 0.5 Units Taq Polymerase (NEB, Frankfurt, Germany), 100 mM dNTPs (Fermentas, St. Leon-Rot, Germany), 20 pmol/μl of each primer, and 1 μg cDNA. Thermal cycling conditions included: pre-cycling step at 95°C for 3 min; 35 cycles of denaturation at 95°C for 45 sec; annealing at 60°C for 45 sec; and extension at 72°C for 90 sec with a final extension step at 72°C for 10 min. All oligonucleotides were designed with an annealing temperature of 60°C. A summary of oligonucleotide sequences, fragment sizes and accession numbers is enlisted in S1 Table.

#### Flow cytometry analysis for MSC markers

Cells were removed from culture dishes, aliquoted equally into fluorescence-activated cell sorting (FACS) tubes (Corning, New York, USA) and stained with corresponding antibodies. Cells incubated with secondary antibody were used as controls. Cells were incubated at room temperature with primary and secondary antibody for 1 hour respectively. After each step, cells were washed twice with PBS and then analyzed with a FACSCalibur™‎ (Becton Dickinson GmbH, Heidelberg, Germany) flow cytometer with a rate of 10,000 events per measurement. Information on primary and secondary antibodies used for flow cytometry experiments is presented in S2 Table.

#### Differentiation potential

Differentiation of MSCs into mesenchymal lineages was achieved by culturing cells in corresponding adipogenic, osteogenic, and chondrogenic differentiation media. For adipogenic differentiation, samples were seeded with a concentration of 5x10^4^ cells/well onto 6-well culture dishes (Cellstar, Greiner BioOne, Frickenhausen, Germany) and cultured for 14 days in DMEM supplemented with 20 mM HEPES zwitterionic buffer (Biochrom AG, Berlin, Germany), 20% FCS HyClone™ (Thermo Fisher Scientific GmbH, Dreieich, Germany), 1 μM dexamethasone (Sigma-Aldrich, St. Louis, USA), 0.5 mM 3-Isobutyl-1-methylxanthin (Sigma-Aldrich, St. Louis, USA), 60 μM indomethacin (Sigma-Aldrich, St. Louis, USA), 1% (v/v) Pen/Strep (Biochrom AG, Berlin, Germany), and 10 μg/ml insulin (Sigma-Aldrich, St. Louis, USA). After 14 days Oil Red O staining was performed for evaluation of lipid vacuoles formation. Briefly, the cells were washed with PBS, fixed for 20 min in 10% formalin (Sigma-Aldrich, St. Louis, USA) and washed twice with ddH_2_O followed by a final wash step with 50% ethanol (AppliChem GmbH, Darmstadt, Germany). Lipid vacuoles were detected after 10 min incubation in Oil Red O (Sigma-Aldrich, St. Louis, USA) in acetone/50% ethanol (Merck KGaA, Darmstadt, Germany) with a final wash step with water and visualized with a bright field microscope (Keyence Biozero, Keyence, Osaka, Japan).

For osteogenic differentiation, samples were seeded with a concentration of 5x10^4^ cells/well onto 6-well culture dishes and cultured for 21 days in DMEM LG (Biochrom AG, Berlin, Germany) supplemented with 20mM HEPES zwitterionic buffer, 10% FCS HyClone™, 0.1 μM dexamethasone, 0.05 mM L-ascorbic acid-2-phosphate (Sigma-Aldrich, St. Louis, USA), 1% (v/v) Pen/Strep and 3 mM Sodium dihydrogen phosphate monohydrate (Carl Roth GmbH, Karlsruhe, Germany). After 21 days, mineralization of the differentiated cells into osteoblasts was detected by Von Kossa staining. In brief, mineralized cells were washed twice with PBS and fixed with 10% formalin, then washed once with PBS and twice with ddH_2_O followed by addition of 1% silver nitrate (Riedel de Haen GmbH, Germany). Afterwards, the plate was exposed to sunlight for 30 min, washed with ddH_2_O, stained for 5 min with 5% sodium thiosulfate (Sigma-Aldrich, St. Louis, USA) and washed again with ddH_2_O. Mineralization was visualized with a bright field Keyence Biozero microscope.

For chondrogenic differentiation, 2.5 × 10^5^ cells/well were pelleted in v-shape 96-well plates (Cellstar, Greiner BioOne, Frickenhausen, Germany) by centrifugation for 5 min at 200 x g. The pellet was incubated for 21 days in DMEM HG culture medium ((Biochrom AG, Berlin, Germany) supplemented with 20 mM HEPES buffer, 1% (v/v) Pen/Strep, 1% (v/v) ITS Universal Cell Culture Supplement Premix (Becton Dickinson GmbH, Heidelberg, Germany), 0.1 μM dexamethasone, 0.17 mM l-ascorbin acid-2-phosphate (Sigma-Aldrich, St. Louis, USA), 0.35 mM l-proline (Biochrom AG, Berlin, Germany), 1 mM sodium pyruvate (Biochrom AG, Berlin, Germany) and 10 ng/ml Transforming Growth Factor-β3 (PeproTech GmbH, Hamburg, Germany). Medium change was performed every 3 days. After 21 days, the pellets were fixed with 10% paraformaldehyde (Carl Roth GmbH, Karlsruhe, Germany), sectioned at 7 μm and stained with Alcian blue (Sigma-Aldrich, St. Louis, USA) as an indicator of sulfated glycosaminoglycan [sGAG]-rich extracellular matrix.

All differentiation procedures were performed in triplicates and compared with undifferentiated cells as controls (n = 3).

### Hypothermic storage of cells

For hypothermic storage, the cell suspension was adjusted to a concentration of 2x10^5^ cells/ml in a culture medium, corresponding to the ratio of the number of cells to the volume of culture medium during cell culturing. Cells were preserved in 10 ml polystyrol tubes with non-adhesive surface (SPL Life Science, Pocheon-Si, South Korea). One portion of the samples was stored at a temperature range from +18°C to +24°C (subnormothermic temperature, which can be considered hypothermic for the cells in comparison to physiological human body temperature), with further evaluation of viability and functional state after 24, 48, 72, and 96 hours. The other portion was stored at +4°C (hypothermic storage), with further evaluation of viability and functional state after 24, 48, 72, and 96 hours. Cells which were directly removed from the culture dish were used as positive controls; cells incubated in 96% ethanol at -20°C were used as negative controls.

### Cryopreservation of cells

#### Analysis of influence of cryoprotective medium

Placental MSCs were equilibrated in cryoprotective medium at concentration of 6x10^5^ cells/ml at +20°C for 15 minutes in 1.8 ml cryogenic vials (Nunc, Thermo Fisher Scientific GmbH, Dreieich, Germany), then cooled in the programmable freezer "ЗП-10" ("BioCold" Special Designing and Technical Bureau, Kharkiv, Ukraine) with a cooling rate of 1°C/min down to -70°C, followed by immersion into liquid nitrogen. Afterwards, samples were stored for 7 days at low-temperature storage tank HB–0.5 (JSC "Ural Compressor Plant", Ekaterinburg, Russia). After low temperature storage the samples were thawed in the water bath at +37°C.

Cryopreservartion medium was composed of cell culture medium containing 10% FBS supplemented with various conventional CPAs in different concentrations: 5% DMSO (Sigma-Aldrich, St. Louis, USA), 10% DMSO, 15% DMSO, 11.4% 1,3-Propanediol (Sigma-Aldrich, St. Louis, USA), 6.8% sucrose (Sigma-Aldrich, St. Louis, USA), 9.3% ethylene glycol (Sigma-Aldrich, St. Louis, USA), 13.8% glycerol (Carl Roth GmbH, Karlsruhe, Germany), 6% HES (Sigma-Aldrich, St. Louis, USA). For selection of a cryoprotective medium with minimal content of substances not authorized in medical practice, the culture medium was replaced with Hank’s solution, Ringer's solution, or 0.9% solution of sodium chloride, all supplemented with 10% DMSO as a CPA. Besides, plasma volume expanders, with main components possessing cryoprotective properties according to the literature [26], were tested as cryopreservation media. These included: 6% dextran (RescueFlow^®^, Pharmanovia, Gentofte, Denmark), sorbitol 6% (Rheosorbilact^®^, YURiA-PHARM Ltd., Kyiv, Ukraine), polyvinylpyrrolidone 6% (Neohemodes^®^, Nicofarm, Kyiv, Ukraine), and 6% hydroxyethyl starch (Refortan^®^ Berlin-Chemie AG, Germany). All tested plasma volume expanding solutions are approved for clinical intravenous administration with doses up to 200–1000 ml, thus, further washing procedures after cryopreservation were not required. Additionally, cryoprotective medium composed of culture medium, containing 10% DMSO, but without FBS, was tested. Non-cryopreserved cells were used as positive controls and cells frozen in culture medium without CPA were used as negative controls.

#### Analysis of influence of cooling rates

Analysis of the influence of cooling rates was performed with cell suspensions in standard culture medium containing 10% FBS and supplemented with 10% DMSO as a CPA. The samples were cooled in the programmable freezer "3П-10" at a cooling rate of 1°C/min to end temperatures -20°C, -30°C, -40°C, -50°C, -60°C, and -70°C, with subsequent immersion into liquid nitrogen, storage for 7 days, followed by thawing in a water bath. Non-cryopreserved cells were used as positive controls and cells cryopreserved with the same cryoprotective medium, but directly immersed into liquid nitrogen were used as negative controls.

Additionally, survival of the cells that have been thawed and re-frozen in the same media without washing and passaging was analyzed.

### Evaluation of cell number, viability and functional activity of cells

The number of cells was counted in a Neubauer hemacytometer (Marienfeld-Superior, Lauda-Königshofen, Germany) by methylene blue staining. Cell viability was determined by staining with 0.4% Trypan blue as well as with flow cytometry method, measured with a FACSCalibur™‎ flow cytometer using 7AAD (Becton Dickinson, Franklin Lakes, USA) according to the manufacturer’s protocol. Data processing of cytofluorimetric analysis was carried out with "Flowing Software" V. 2.1 program.

Adhesive properties were studied by passaging 2x10^5^ cells per well on a 6-well plate, and culturing in a humidified CO_2_ incubator (Thermo Fisher Scientific GmbH, Dreieich, Germany) at 5% CO_2_ and 37°C. On the following day non-adhered cells were washed off and remaining adhered cells were detached with 0.25% trypsin solution and counted.

Cultural properties were studied by passaging 2x10^5^ cells per well on a 6-well plate, and culturing in a humidified CO_2_ incubator at 5% CO_2_ and 37°C until monolayer formation.

Metabolic activity of the cells was studied with application of MTT test, CellTiter 96^®^ Non-Radioactive Cell Proliferation Assay (Promega, Fitchburg, USA), according to the manufacturer’s protocol. In brief, 1 x 10^4^ cells per well were seeded on a 96-well plate (Cellstar, Greiner BioOne, Frickenhausen, Germany), with four parallels for each sample, and cultivated for 24 hours in 100 μl MSC medium in CO_2_ incubator at 37°C and 5% CO_2_. After 24-hour incubation, 15 μl MTT reagent per well were added, following incubation for 4 hours at 37°C. After incubation 100 μl Stop Mix reagent per well were added with further incubation for 1 hour at room temperature in the absence of light. Afterwards, the formazan concentration was measured at a wavelength of 570 nm with a BioRad 680 microplate reader (BioRad, Munich, Germany).

Resazurin reduction test was performed by plating 1 ml of culture medium with cells in concentration of 1x10^5^ cells/ml into each well of the 24-well plates (SPL Life Science, Pocheon-Si, South Korea) with the following addition of 200 μl of resazurin solution (Sigma-Aldrich, St. Louis, USA) in PBS in concentration of 0.15 mg/ml with further incubation for 24 hours, as described before. The absorption was measured with a PV 1251C spectrophotometer (Solar, Minsk, Belarus) at a wavelength of 570 nm.

### Data analysis and statistics

All data was collected in triplicates from 3 to 5 independent experiments (n≥3). Mean values and standard deviation calculations (mean±SD) with the following analysis with Mann-Whitney criteria and Fisher's method were used to obtain statistically significant conclusions. The changes of parameters were considered statistically significant at p<0.05. Statgraphics V 2.1 software was utilized for statistical calculations and data analysis.

## Results and Discussion

### Characterization of the MSCs

**Fig 1:**
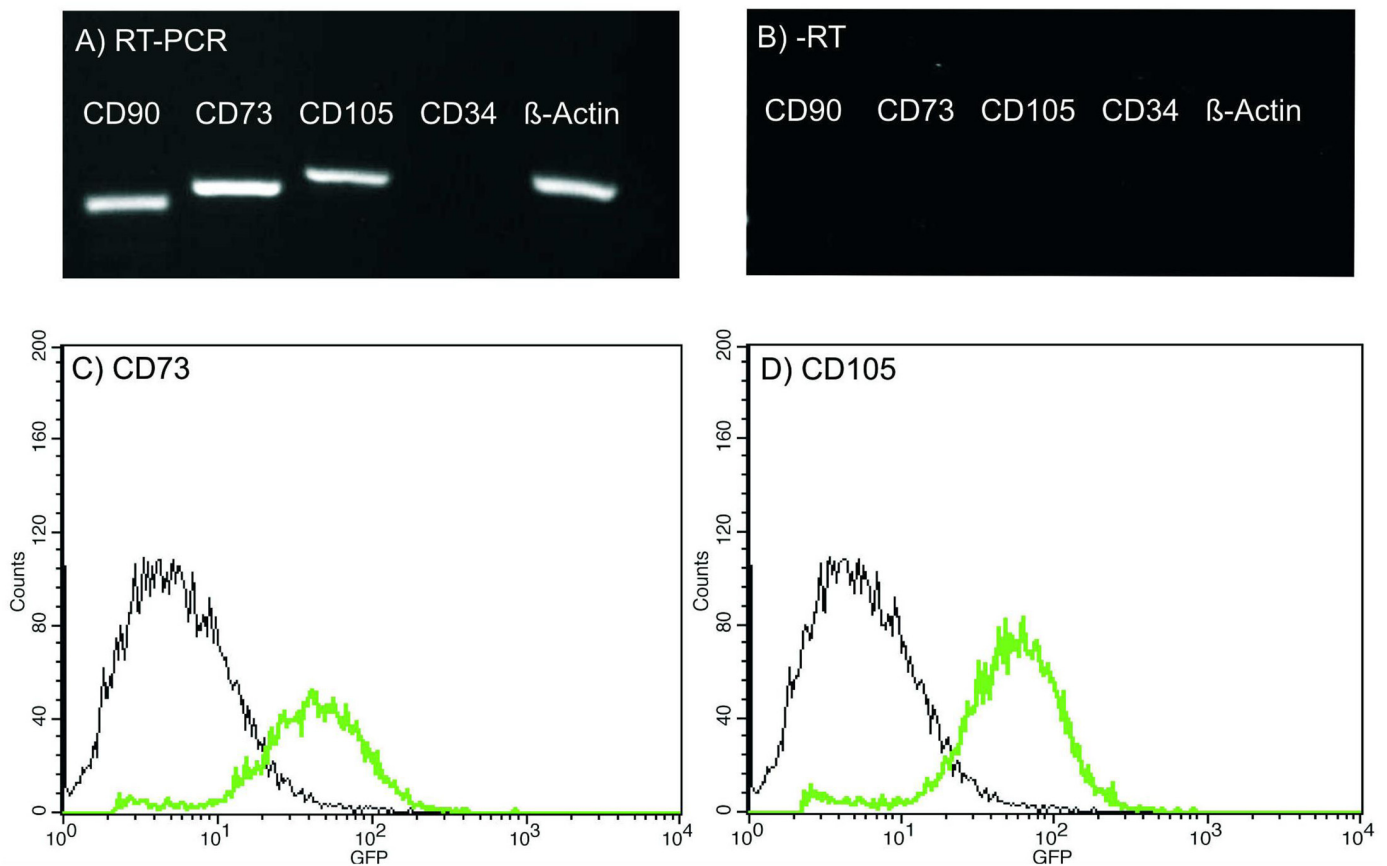
Characterization of primary cell culture derived from placenta for MSC origin. (A) RT-PCR analysis for expression of typical MSC markers: cells provide a signal for CD90, CD73, and CD105 MSC while being negative for CD34 hematopoietic marker. (B) Minus RT control for RT-PCR. (C,D) Cells display strong signals for CD73 and CD105 in flow cytometry analysis.

**Fig 2:**
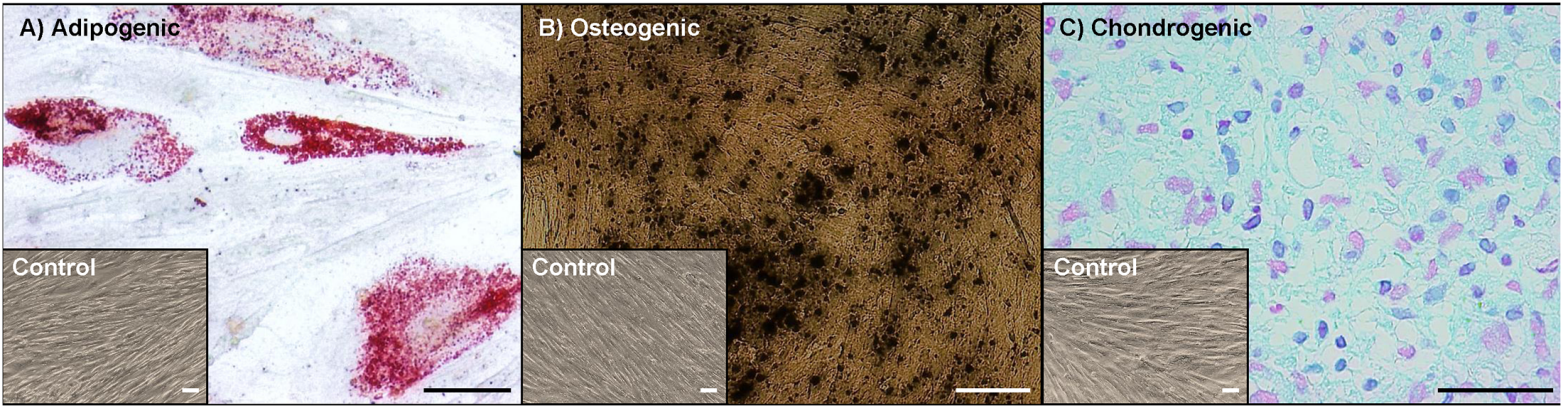
Differentiation potential of primary cell culture derived from placenta into lineages of MSC origin. MSCs were differentiated into (A) adipogenic (Oil Red O staining), (B) osteogenic (Von Kossa staining), and (C) chondrogenic (Alcian blue staining) lineages as a proof of principle for plasticity of MSCs. Cells expanded in regular MSC culture medium represent the negative control (bar = 50 μm).

Primary cells were adherent to plastic, displayed typical spindle-shaped fibroblast-like morphology in cell culture conditions and were capable to form a monolayer. RT-PCR analysis revealed that cells were positive for typical MSC markers, such as CD90, CD73, and CD105 and negative for hematopoietic marker CD34 (Fig 1A and 1B). These results were also confirmed on protein level by flow cytometry analysis for CD73 and CD105 markers (Fig 1C and 1D). Besides, the cells were capable of differentiating into adipogenic, chondrogenic, and osteogenic mesenchymal lineages (Fig 2). Listed features are considered sufficient for characterizing a cell line as MSC [25,27].

### Hypothermic and subnormothermic temperature storage

**Fig 3:**
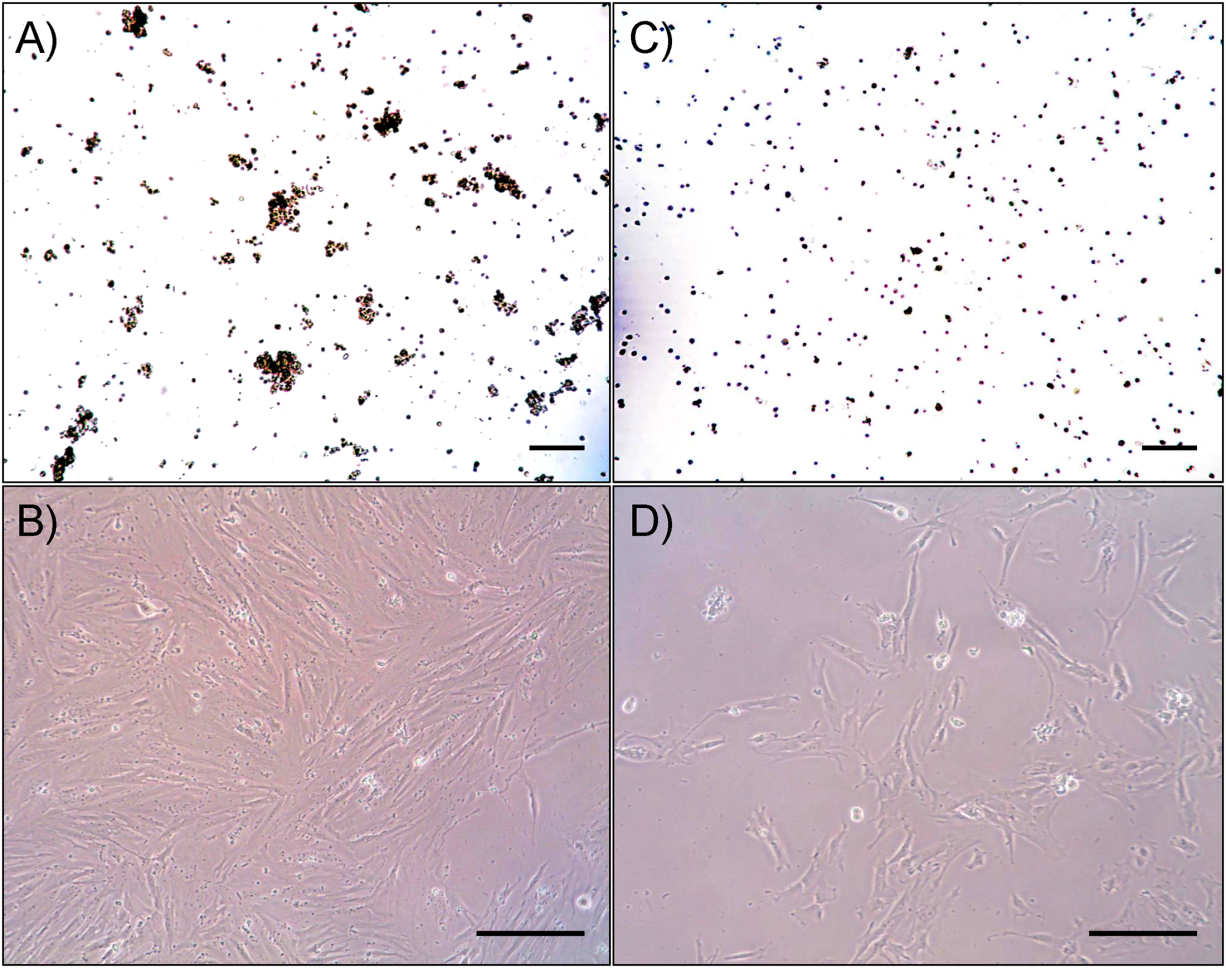
MSCs of placenta after storage at +20°C (A,B) and 4°C (C,D). Trypan blue staining, (A,C); phase contrast microscopy (B,D). Scale bar is 200 μm.

The cell suspension formed aggregates of up to 50–200 μm (Fig 3a) with a minor percentage of single cells when kept at +20°C overnight. There was an average 10% decrease of counted cells (1.8- 2x10^5^ cells/ml, Table 1) with a decrease of viability down to 82.9±5.5% when monitored by Trypan blue staining. Monolayer was formed on the 2nd day (Fig 3b). After 48 hours of subnormothermic storage a slight decrease in cell number, viability and adhesive properties was observed, but cell conglomerates remained of the same size and number as before; a monolayer formed on day 3–4. After 72-hour storage the cell concentration dropped down to 1.5x10^5^ cells/ml, viability decreased to 69.3±5.4% and the monolayer was formed on the 4th day. Further storage of cell suspension under these conditions led to drastic decrease in viability and incapability of monolayer formation.

**Table 1:** Characteristics of placental MSCs after short-term storage at different temperature conditions (mean±SD).

	Cell number, *х 10* ^*5*^	Viability by Trypan Blue staining, *%*	Adhered cells, *%*	Monolayer formation, *days*
**Control**	2.1±0.2	95.3±1.8	95.2±1.5	1
**Cryopreservation**	2.0±0.25	86.2±7.4	81.3±7.3	2
**Storage at +20°C**				
**24 hours**	2.0±0.1	82.9±5.5	80.4±6.4	2
**48 hours**	1.8±0.06	78.5±6.8	71.2±5.5	3
**72 hours**	1.5±0.05*	69.3±5.4*	65.2±4.4*	4
**96 hours**	1.2±0.05*	72.5±6.8*	60.5±5.8*	-
**Storage at +4°C**				
**24 hours**	0.9±0.08*	73.8±5.4*	51.2±3.5*	8
**48 hours**	0.4±0.05*	50.5±5.6*	52.5±5.9*	-
**72 hours**	0.2±0.04*	45.3±4.5*	50.2±4.3*	-

*- significance to control p<0.05.

Analysis of cellular characteristics after hypothermic storage at +4°C in the culture medium without additional components for 24 hours revealed significant decrease of the cell number (Table 1), which corresponds with the literature [28,29]. Interestingly, all cells were distinctly located without forming conglomerates (Fig 3c) and adhered instead with poor distribution on the dish, slow proliferation and late monolayer formation on day 8 (Fig 3d). Hypothermic storage of cells for more than 24h resulted in significant decrease of viability, abolishing all further attempts of cell culture.

Several research groups have demonstrated the option of short term storage of stem cells and other cell types at room temperature (subnormothermic) conditions [20,30,31]. In this study, we could confirm storage of the cells for up to 3 days preserving cell number, viability and cellular properties. Therefore we conclude it is convenient to use this short-term storage condition (+20°C) e.g. for transport purposes or for short term storage prior analysis in hospitals and diagnostic laboratories.

As to hypothermic storage, different levels of success can be found in the literature, strongly depended on the cell type and composition of preservation media [19,32,33]. In summary, hypothermic storage of cells in suspension causes swelling of cells due to cold inactivation of membrane pumps, as well as activation of cellular stress pathways [28,34] resulting in rapid depletion of cellular energy reserves and loss of stored material. For our studied cell type, hypothermic storage in culture medium without the addition of colloids, antioxidants, and sugars does not allow necessary preservation outcome during this period, which is most likely connected to damages caused by hypothermia.

### Cryopreservation

#### Influence of cryoprotective medium

Cryopreservation of cells with 5%, 10%, or 15% DMSO did not result in significant difference from the positive control in terms of cell number and cell viability according to Trypan blue staining, however, flow cytometry analysis with 7AAD showed minor reduction in viability in samples frozen with 15% DMSO. Metabolic activity data via MTT test also showed no differences compared to positive control. However, cell adherence decreased with lowering of DMSO concentration to 5% as well as monolayer formation was observed not on the 2nd day, as with the other samples, but on the 3rd day (Table 2).

**Table 2:** Cryopreservation of placental MSCs with various CPAs (mean±SD).

	Cell number,*х 10* ^*5*^	Viability by Trypan Blue, *%*	7AAD, *%*	MTT, *absorbtion at 570 nm*	Adhered cells, *%*	Monolayer formation, *days*
**Negative control (no CPA)**	5.5±0.4	5.2±3.7	89.1±8.8	0.235 ±0.02	-	-
**Positive control (native cells)**	6.0±0.5	95.4±2.2	8.3±6.5	0.783 ±0.05	92.2±5.6	2
**15% DMSO**	5.9±0.3	89.0±5.1	22.2±1.9*	0.798 ±0.06	85.5±8.3*	2
**10% DMSO**	5.8±0.4	93.9±3.1	16.5±0.9*	0.887 ±0.07	89.5±5.6	2
**5% DMSO**	5.9±0.5	93.8±4.9	15.8±1.3*	0.878 ±0.08	75.6±8.1	3
**11.4% Propanediol**	6.0±0.7	92.7±3.6	20.3±2.1*	0.989 ±0.1	65.8±7.6*	3
**6.8% Sucrose**	5.7±0.3	58.4±3.6*	65.7±5.8*	0.546 ±0.04*	59.5±4.5*	5
**9.3% Ethylene glycol**	4.1±0.2*	85.6±7.8	18.6±3.0*	1.013 ±0.09*	66.5±5.4*	3
**13.8% Glycerol**	5.8±0.3	83.4±5.7	45.9±3.6*	0.539 ±0.04*	42.5±3.8*	4

*- significance to positive control p<0.05.

While after cryopreservation with propanediol the cell number and viability were not significantly changed according to Trypan blue staining, analysis with 7AAD in flow cytometry showed a decrease of viability. Here, MTT test showed an increase of activity, while at the same time adherence decreased to 65.8%±7.6 and monolayer was formed on the 3rd day.

Although after cryopreservation with sucrose the cell number remained unchanged, their viability dropped significantly to 58.4%±3.6, according to Trypan blue and 65.7%±5.8 of cells were positively stained for 7AAD. The level of MTT was reduced almost two fold as well as adherence, while monolayer was formed late at day 5.

Cryopreservation with ethylene glycol resulted in decrease of the cell number with preservation of viability, accompanied by increasing levels of MTT. However, only 66.5%±5.4 of cells adhered and monolayer was formed on the 3rd day.

Application of glycerol as a CPA did not result in decrease of the cell number and allowed preserving the viability. The level of MTT was lowered, but only 42.5%±3.8 of cells adhered and monolayer was formed on the 4th day.

Cryopreservation of samples in culture medium without the addition of serum did not result in any significant differences in comparison to the samples containing serum in all of the studied parameters. When preserved with 100% serum, the number of cells and their viability reduced; 81.5%±5.6 were capable to adhere; and the cells formed a monolayer 24h later (Table 3).

**Table 3:** Cryopreservation of placental MSCs with various cryoprotective media (mean±SD).

	Cell number, *х 10* ^*5*^	Viability by Trypan Blue, *%*	7 AAD, *%*	MTT, *absorbtion at 570 nm*	Adhered cells, *%*	Monolayer formation, *days*
**Negative control (no CPA)**	5.5±0.4	5.2±3.7	89.1±8.8	0.235 ±0.02	-	-
**Positive control (native cells)**	6.0±0.5	95.4±2.2	8.3±6.5	0.783 ±0.05	92.2±5.6	2
**10% DMSO+culture medium (no serum)**	6.0±0.4	94.1±2.4	12.1±1.1	0.852 ±0.04	90.5±8.1	2
**Serum +10% DMSO**	4.4±0.2*	83.4±4.3*	22.5±2.1*	0.823 ±0.05	81.5±5.6*	3
**0,9% NaCl + 10% DMSO**	2.9±0.2*	75.2±6.5*	58.6±5.2*	0.497 ±0.03*	72.1±6.5*	6
**Henks solution +10% DMSO**	3.8±0.5*	82.3±6.4*	33.5±2.4*	0.668 ±0.02*	88.5±6.3	3
**Ringer's solution + 10% DMSO**	3.2±0.2*	82.5±8.1*	34.9±3.5*	0.836 ±0.05	95.2±7.2*	3
**6% Dextran**	4.1±0.4*	93.2±5.6	32.5±2.3*	0.658 ±0.05*	83.2±7.5*	3
**6% PVP**	5.1±0.6*	91.2±9.5	28.2±3.5*	0.635 ±0.04*	84.5±7.6	3
**6%Sorbitol**	3.4±0.4*	17.2±1.2*	67.3±6.8*	0.325 ±0.02*	15.3±2.1*	8
**6% HES**	1.5±0.2*	20.9±2.2*	65.5±5.4*	0.360 ±0.02*	29.2±2.1*	10

*- significance to positive control p<0.05.

Experiments on replacement of culture medium with crystalloid solutions accepted for clinical applications, revealed the optimal results with Ringer's and Hank’s solutions, which are the most physiologically balanced in salt content and pH (Table 3). Their application resulted in 30% reduction of the cell number and about 20–30% decrease of viability, depending on the method of analysis. Interestingly, the cellular adhesion capability remained at quite high level allowing monolayer formation on day 3. However, cryopreservation in medium containing only 0.9% sodium chloride with 10% DMSO led to decrease of cell number to roughly 50%, the viability decreased to 42–75% and a monolayer was formed late at day 6. Attempts to replace the cryopreservation medium with 6% dextran or 6% polyvinylpyrrolidone (PVP) without adding DMSO resulted in significant decrease of the cell number, but remained cells retain viability and adhesive properties and formed a monolayer at day 3–4. After application of 6% Sorbitol or 6% HES the number of cells was reduced by more than two fold, adhesive properties were drastically reduced and monolayer was formed only on 8–10th day.

Negative controls were frozen in complete culture medium without addition of CPAs, while certain other samples were frozen in pharmacopoeia solutions where pH level, osmolality, ionic, vitamin and amino acid composition may differ from such parameters of the cell culture medium. This could explain a slightly higher cell count in negative controls after cryopreservation in comparison to the samples frozen without complete cell culture medium (Table 3).

In this study, our results displayed that DMSO, propanediol and ethylene glycol were the most effective among studied CPAs for the placental MSCs. Interestingly, as to the other components of the medium, additional serum appeared to be not necessary for cryopreservation outcome within the studied parameters; the highest survival of cells could be observed in serum-free standard culture medium containing CPAs. Furthermore, it is possible to replace the culture medium with Ringer’s or Hank’s solution with a slight decrease in the cell number and viability parameters after cryopreservation, which become significantly reduced in case of replacing the culture medium with sodium chloride physiological solution. Such results indicate possibilities for the future towards development of serum- and xeno-free cryopreservation techniques currently investigated by other research groups [35,36] complying with latest GMP standards. Besides, we demonstrate the new possibility to use dextran or polyvinylpyrrolidone in cryopreservation solutions, which also possess cryoprotective effects, but cause the reduction of cell number and viability by 20–30%. Application of sorbitol or HES drastically reduces these parameters and is inconvenient. Other groups were able to show efficiency of HES only in combination with other CPAs, but not the HES alone [37].

#### Influence of cooling rates

**Fig 4:**
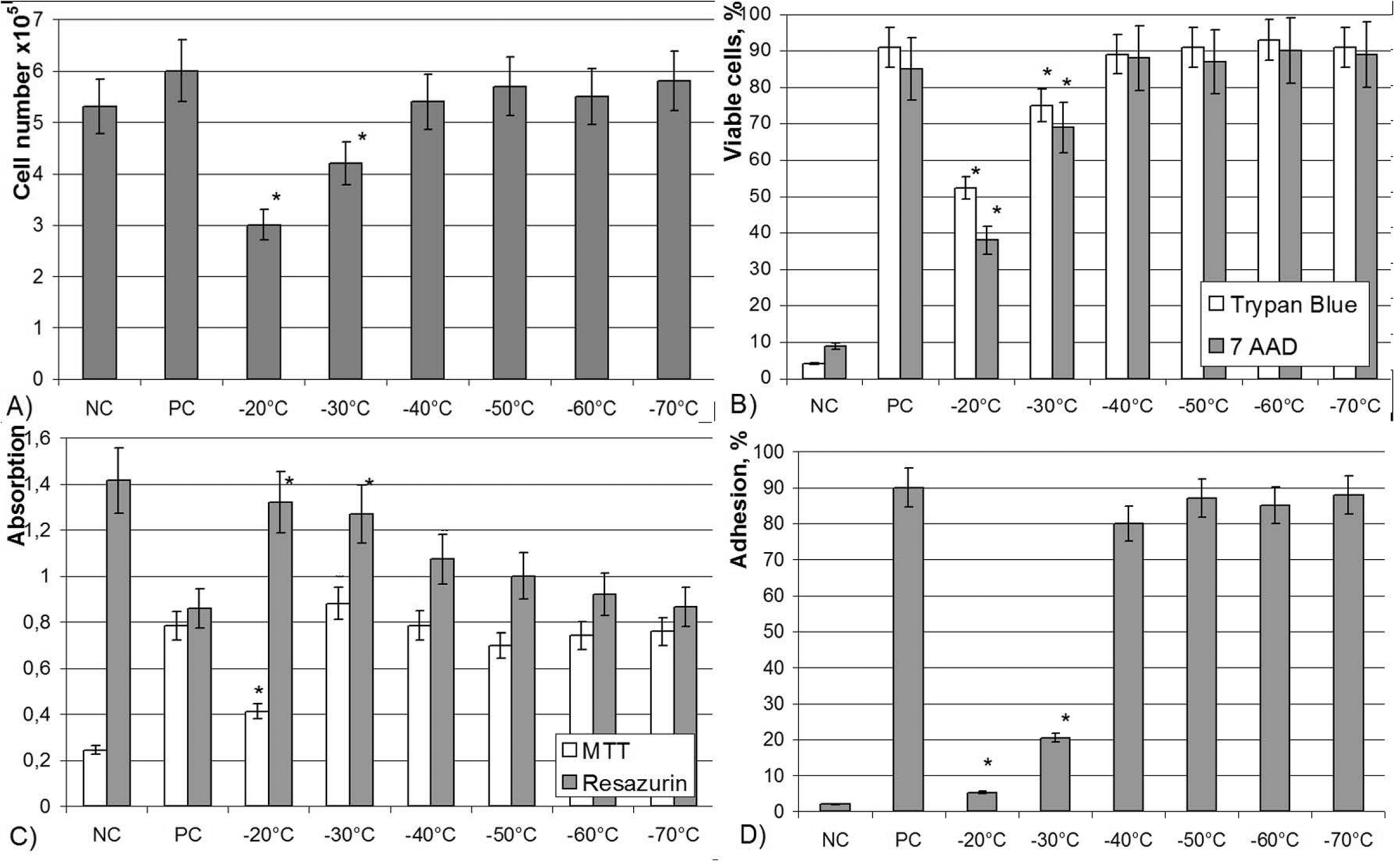
Parameters of placental MSCs after cryopreservation with different cooling rates. (A) - cell number in the sample; (B) - viable cells according to Trypan blue and 7 AAD staining; (C) - functional activity of cells in studied samples; (D) - adherence to plastic. *- significance to positive control p<0.05.

One of the key parameters influencing the outcome of cryopreservation is the cooling rate [23]. In general, freezing protocols for MSCs seem to be reasonably established [24], but it is still necessary to analyze other options, which might be convenient from the practical and economic perspectives for a particular cell type. Placental MSC number decreased only slightly, to 5.3 x 10^5^ after cooling by direct immersion into liquid nitrogen. However, only 2.3–9% of these cells still possessed viability, values of MTT test or resazurin reduction were below detection limits and only few single cells adhered to plastic (Fig 4). Thus, formation of a monolayer was not observed (Table 4).

**Table 4:** Terms of monolayer formation in the studied samples.

	Negative control	Positive control	-20°C	-30°C	-40°C	-50°C	-60°C	-70°C
**Days**	0	2	0	6	2	2	2	2

Slow cooling to -20°C decreased cell number and viability to about 50% of the cells initially frozen. Whereas measurable data of resazurin reduction and MTT test were obtained, only single cells were capable to adhere (Fig 4) and were not sufficient to form a monolayer (Table 4).

Cooling down to -30°C allowed survival of significantly larger number of cells: about 60%, with 70–80% of them being viable. Functional tests were positive, about 20% of cells adhered to plastic (Fig 4) and formed a monolayer on the 6th day in culture (Table 4).

Pronounced increase in MTT reaction alongside the decrease of resazurin reduction may be explained by necrotic and apoptotic changes in the cells after thawing [38] in the first hours, and their death within one day, since the MTT test reaction occurs within 4 hours, while resazurin reduction test that we used lasts 24 hours.

Cooling down to the range between -40°C and -80°C allowed to preserve cell number along with parameters of viability, functional tests and time required for the formation of monolayer without significant differences between the samples. An observed decrease of cell number and their functional characteristics at -40°C was not statistically significant.

In summary, our study demonstrates -40°C to -50°C as the minimum required end temperature to which placental MSC samples should be slowly cooled for short term cryopreservation, preferably transferred into liquid nitrogen for long term storage. Besides, from economical point of view, such parameters allow application of refrigeration equipment which is about 10 times less expensive in cost of acquisition and energy than the -80°C refrigerators and thus, could be available in a wider range of medical institutions and blood transfusion centers. Furthermore, establishment of -40/50°C storage protocols might lead to disposal of expensive programmable freezer technology and exclusion of costs for liquid nitrogen, if long term storage is not necessary.

When re-freezing placental MSC in the same medium directly after thawing, a twofold decrease down to the total number of cells to 2.5±0.5х10^5^ cells/ml was observed; viability was approximately 81.2±8.3% according to Trypan blue staining and 63.7% in the study by flow cytometry with 7AAD, while activity according to MTT test was 0.923±0.09; about 50% of cells have adhered to plastic and monolayer was formed in 3–4 days.

The findings suggest that repeated freezing—thawing with a standard protocol drastically reduces cell number and viability, while the remained MSCs retain cellular and metabolic properties.

## Conclusions

Short-term storage of placental MSCs at subnormothermic condition ensured the preservation of cell number, viability and cultural properties within 48 hours whereas further increase of the storage period at this temperature led to significant loss of both cell number and viability. The maximum period of storage at this temperature with possibility of cellular recovery and a monolayer formation was 72 hours.

Hypothermic storage at +4°C in cell culture medium without additional modifications led to reduction of the cell number and compromised cultural properties within 24 hours without chances of recovery.

DMSO and propandiol were the optimal CPAs for cryopreservation of placental MSCs ensuring maximum cell viability and retention of metabolic and cultural properties. The key role belongs to the ionic composition and acid-base balance of the cryoprotective medium, while adding extra serum did not significantly affect the outcome of cryopreservation.

In order to comply with the GMP requirements it is possible to cryopreserve placental MSCs without the standard culture medium by maintaining ionic composition and application of dextran or polyvinylpyrrolidone, which are certified for clinical use. However, the cell number and cell viability decreased by 20–30% after such cryopreservation procedures.

For cryopreservation of placental MSCs under GMP requirements it was possible to exclude serum without viability alteration. Moreover, it is possible to replace the culture medium for the Ringer's or Hank's solution (HBSS), although accompanied with the loss of approximately 20% of cells and a slight decrease in cell viability.

The optimal cooling rate for cryopreservation of placental MSCs was 1°C/min down to -40°C, followed by immersion into liquid nitrogen. Increasing of this temperature led to significant loss in cell number and viability with the upper temperature limit preceding rapid cooling (immersion to LN) and allowing further culture recovery at -30°C. Interestingly, lowering the temperature to -80°C did not result in any significant alterations.

Re-freezing of placental MSCs directly after thawing in the same medium without recultivation led to 50% loss of the cell number and 20% decrease of viability.

In summary, the data obtained in this study indicates the necessity and perspectives of development of alternative cryopreservation protocols for clinical application of MSCs utilizing GMP approved components and certified equipment, available or affordable to modern hospitals.

## Supporting Information

S1 Table Oligonucleotides with corresponding accession numbers. (DOC)

S2 Table Antibodies used with corresponding working dilutions and origin. (DOC)

## References

[1] Bárcena A, Muench MO, Kapidzic M, Gormley M, Goldfien GA, Fisher SJ (2011) Human placenta and chorion: potential additional sources of hematopoietic stem cells for transplantation. Transfusion 51 Suppl 4:94S–105S. 10.1111/j.1537-2995.2011.03372.x 22074633 PMC3266842

[2] Pipino C, Shangaris P, Resca E, Zia S, Deprest J, Sebire NJ, et al (2013) Placenta as a reservoir of stem cells: an underutilized resource? Br Med Bull 105:43–68. 10.1093/bmb/lds033 23184854

[3] Hass R, Kasper C, Böhm S, Jacobs R (2011) Different populations and sources of human mesenchymal stem cells (MSC): A comparison of adult and neonatal tissue-derived MSC. Cell Commun Signal 9:12 10.1186/1478-811X-9-12 21569606 PMC3117820

[4] Leeb C, Jurga M, McGuckin C, Moriggl R, Kenner L (2010) Promising new sources for pluripotent stem cells. Stem Cell Rev 6(1):15–26. 10.1007/s12015-009-9102-0 20091142

[5] Park S, Koh SE, Hur CY, Lee WD, Lim J, Lee YJ (2013) Comparison of human first and third trimester placental mesenchymal stem cell. Cell Biol Int 37(3):242–249. 10.1002/cbin.10032 23364891

[6] Stubbendorff M, Deuse T, Hua X, Phan TT, Bieback K, Atkinson K, et al (2013) Immunological properties of extraembryonic human mesenchymal stromal cells derived from gestational tissue. Stem Cells Dev 22(19):2619–2629. 10.1089/scd.2013.0043 23711207

[7] Kim YH, Park TC, Lee G, Shin JC (2012) Gene expression pattern of human chorion-derived mesenchymal stem cells during adipogenic differentiation. Yonsei Med J 53(5):1036–1044. 10.3349/ymj.2012.53.5.1036 22869490 PMC3423845

[8] Murphy MB, Moncivais K, Caplan AI (2013) Mesenchymal stem cells: environmentally responsive therapeutics for regenerative medicine. Exp Mol Med 15;45:e54. 10.1038/emm.2013.94 PMC3849579 24232253

[9] Seo MS, Park SB, Kim HS, Kang JG, Chae JS, Kang KS (2013) Isolation and characterization of equine amniotic membrane-derived mesenchymal stem cells. J Vet Sci 14(2):151–159. 23388430 10.4142/jvs.2013.14.2.151 PMC3694186

[10] Vellasamy S, Sandrasaigaran P, Vidyadaran S, George E, Ramasamy R (2012) Isolation and characterisation of mesenchymal stem cells derived from human placenta tissue. World J Stem Cells. 4(6):53–61. 22993662 10.4252/wjsc.v4.i6.53 PMC3443712

[11] Kesting MR, Loeffelbein DJ, Steinstraesser L, Muecke T, Demtroeder C, Sommerer F, et al (2008) Cryopreserved human amniotic membrane for soft tissue repair in rats. Ann Plast Surg 60(6):684–691. 10.1097/SAP.0b013e31814fb9d2 18520208

[12] Cirelli N, Lebrun P, Gueuning C, Moens A, Delogne-Desnoeck J, Dictus-Vermeulen C, et al (2000) Secretory characteristics and viability of human term placental tissue after overnight cold preservation. Hum Reprod 15(4):756–761. 10739815 10.1093/humrep/15.4.756

[13] Luetzkendorf J, Nerger K, Hering J, Moegel A, Hoffmann K, Hoefers C, et al (2015) Cryopreservation does not alter main characteristics of Good Manufacturing Process-grade human multipotent mesenchymal stromal cells including immunomodulating potential and lack of malignant transformation. Cytotherapy 17(2):186–198. 10.1016/j.jcyt.2014.10.018 25593077

[14] Holm F, Ström S, Inzunza J, Baker D, Strömberg AM, Rozell B, et al (2010) An effective serum- and xeno-free chemically defined freezing procedure for human embryonic and induced pluripotent stem cells. Hum Reprod 25(5):1271–1279. 10.1093/humrep/deq040 20208061 PMC2854046

[15] Kocaoemer A, Kern S, Klüter H, Bieback K (2007) Human AB serum and thrombin-activated platelet-rich plasma are suitable alternatives to fetal calf serum for the expansion of mesenchymal stem cells from adipose tissue. Stem Cells 25(5):1270–1278. 17255520 10.1634/stemcells.2006-0627

[16] Minor T, Paul A (2013) Hypothermic reconditioning in organ transplantation. Curr Opin Organ Transplant 18(2):161–167. 10.1097/MOT.0b013e32835e29de 23313939

[17] Guibert EE, Petrenko AY, Balaban CL, Somov AY, Rodriguez JV, Fuller BJ (2011) Organ Preservation: Current Concepts and New Strategies for the Next Decade. Transfus Med Hemother 2011;38(2):125–142. 21566713 10.1159/000327033 PMC3088735

[18] Parsons RF, Guarrera JV (2014) Preservation solutions for static cold storage of abdominal allografts: which is best? Curr Opin Organ Transplant 19(2):100–107. 10.1097/MOT.0000000000000063 24553501

[19] Corwin WL, Baust JM, Baust JG, Van Buskirk RG (2013) Implications of differential stress response activation following non-frozen hepatocellular storage. Biopreserv Biobank 11(1):33–44. 10.1089/bio.2012.0045 24845253 PMC4076978

[20] van de Kerkhove MP, Hoekstra R, van Nooijen FC, Spoelstra FO, Doorschodt BM, van Wijk AC, et al (2006) Subnormothermic preservation maintains viability and function in a porcine hepatocyte culture model simulating bioreactor transport. Cell Transplant 15(2):161–8. 16719049 10.3727/000000006783982089

[21] Liu Q, Berendsen T, Izamis ML, Uygun B, Yarmush ML, Uygun K (2013) Perfusion defatting at subnormothermic temperatures in steatotic rat livers. Transplant Proc 45(9):3209–13. 10.1016/j.transproceed.2013.05.005 24182786 PMC3843811

[22] Thirumala S, Goebel WS, Woods EJ (2009) Clinical grade adult stem cell banking. Organogenesis 5(3):143–154. 20046678 10.4161/org.5.3.9811 PMC2781095

[23] Mazur P (2010) A biologist's view of the relevance of thermodynamics and physical chemistry to cryobiology. Cryobiology 60(1):4–10. 10.1016/j.cryobiol.2009.12.001 19962974 PMC3733660

[24] Thirumala S, Goebel WS, Woods EJ (2013) Manufacturing and banking of mesenchymal stem cells. Expert Opin Biol Ther 13(5):673–691. 10.1517/14712598.2013.763925 23339745

[25] Pogozhykh O, Pogozhykh D, Neehus AL, Hoffmann A, Blasczyk R, Müller T (2015) Molecular and cellular characteristics of human and non-human primate multipotent stromal cells from the amnion and bone marrow during long term culture. Stem Cell Res Ther. 22;6(1):150 10.1186/s13287-015-0146-6 26297012 PMC4546288

[26] Fuller BJ (2004) Cryoprotectants: the essential antifreezes to protect life in the frozen state. Cryo Letters 25(6):375–388. 15660165

[27] Dominici M, Le Blanc K, Mueller I, Slaper-Cortenbach I, Marini F, Krause D, et al (2006) Minimal criteria for defining multipotent mesenchymal stromal cells. The International Society for Cellular Therapy position statement. Cytotherapy 8(4):315–317. 16923606 10.1080/14653240600855905

[28] Corwin WL, Baust JM, Baust JG, Van Buskirk RG (2014) Characterization and modulation of human mesenchymal stem cell stress pathway response following hypothermic storage. Cryobiology 68(2):215–226. 10.1016/j.cryobiol.2014.01.014 24508650 PMC4001798

[29] Shevchenko MV, Sukach AN (2012) Effect of Hypothermic Storage of Isolated Neural Cells of Newborn Rats in Media of Different Composition on Its Behavior in Culture. Problems of cryobiology 22(4):423–432.

[30] Pereira-Cunha FG, Duarte AS, Reis-Alves SC, Olalla Saad ST, Metze K, Lorand-Metze I, et al (2014). Umbilical cord blood CD34(+) stem cells and other mononuclear cell subtypes processed up to 96 h from collection and stored at room temperature maintain a satisfactory functionality for cell therapy. Vox Sang 108(1):72–81. 10.1111/vox.12199 25333825

[31] Eckstein M, Zimmermann R, Roth T, Hauck-Dlimi B, Strasser EF, Xiang W (2015) The effects of an overnight holding of whole blood at room temperature on haemoglobin modification and in vitro markers of red blood cell aging. Vox Sang 108(4):359–367. 10.1111/vox.12235 25753392

[32] Wright GJ, Brockbank KG, Rahn E, Halwani DO, Chen Z, Yao H (2014) Impact of storage solution formulation during refrigerated storage upon chondrocyte viability and cartilage matrix. Cells Tissues Organs 199(1):51–58. 10.1159/000363134 25171188 PMC4184947

[33] Brockbank KG, Rahn E, Wright GJ, Chen Z, Yao H (2011b) Impact of hypothermia upon chondrocyte viability and cartilage matrix permeability after 1 month of refrigerated storage. Transfus Med Hemother 38(6): 387–392. 22403523 10.1159/000334595 PMC3268002

[34] Baust JM, Snyder KK, VanBuskirk RG, Baust JG (2009) Changing paradigms in biopreservation. Biopreserv Biobank 7(1):3–12. 10.1089/bio.2009.0701.jmb 24845765

[35] Heathman TR, Glyn VA, Picken A, Rafiq QA, Coopman K, Nienow AW, et al (2015) Expansion, harvest and cryopreservation of human mesenchymal stem cells in a serum-free microcarrier process. Biotechnol Bioeng 112(8):1696–707. 10.1002/bit.25582 25727395 PMC5029583

[36] Al-Saqi SH, Saliem M, Quezada HC, Ekblad Å, Jonasson AF, Hovatta O, et al (2014) Defined serum- and xeno-free cryopreservation of mesenchymal stem cells. Cell Tissue Bank 16(2):181–93. 10.1007/s10561-014-9463-8 25117730

[37] Orellana MD, De Santis GC, Abraham KJ, Fontes AM, Magalhães DA, Oliveira VC, et al (2015) Efficient recovery of undifferentiated human embryonic stem cell cryopreserved with hydroxyethyl starch, dimethyl sulphoxide and serum replacement. Cryobiology S0011-2240(15)00023-1. 10.1016/j.cryobiol.2015.01.005 25641609

[38] Lund PK, Westvik AB, Joø GB, Øvstebø R, Haug KB, Kierulf P (2001) Flow cytometric evaluation of apoptosis, necrosis and recovery when culturing monocytes. J Immunol Methods 252(1–2):45–55. 11334964 10.1016/s0022-1759(01)00330-1

