# Supplementary material for: Influence of Factors of Cryopreservation and Hypothermic Storage on Survival and Functional Parameters of Multipotent Stromal Cells of Placental Origin
Source: PLoS One. 2015 Oct 2;10(10):e0139834. doi: 10.1371/journal.pone.0139834 (PMC4592233; doi:10.1371/journal.pone.0139834)
Supplement: S1 Table — (DOC) [file pone.0139834.s001.doc]

| **Gene** | **Sequence** | **Fragment size (bp)** | **Accession number** |
| --- | --- | --- | --- |
| **CD 90** | 5´- TCC CAG AAC GTC ACA GTG CT - 3´ 5´- AGG GAC ATG AAA TCC GTG GC - 3´ | 134 | NM_006288.3 |
| **CD 73** | 5´- ATG GCT CCT CTC AAT CAT GC - 3´ 5´- ATC AAT GGG CGA CCG GAT AC - 3´ | 158 | NM_001204813.1 |
| **CD 105** | 5´- AGG CCC TGG GAA TCC CAC TG – 3 5´- GGA TGC TCT GGG GGT CAT TC - 3´ | 180 | NM_001114753.2 |
| **CD 34** | 5´- AAT CTG ACC TGA AAA AGC TG - 3´ 5´- ACC GTT TTC CGT GTA ATA AG - 3´ | 212 | NM_001025109.1 |
| **ß-Actin** | 5´- CAT CAT CAC CAA CTG GGA C - 3´ 5´ - GAT AGC CAC ATA CAT GGC TG - 3´ | 187 | NM_001613.2 |
