# Supplementary material for: Influence of Factors of Cryopreservation and Hypothermic Storage on Survival and Functional Parameters of Multipotent Stromal Cells of Placental Origin
Source: PLoS One. 2015 Oct 2;10(10):e0139834. doi: 10.1371/journal.pone.0139834 (PMC4592233; doi:10.1371/journal.pone.0139834)
Supplement: S2 Table — (DOC) [file pone.0139834.s002.doc]

| **CD antigen** | **Company** | **Cat N** | **Dilution** |
| --- | --- | --- | --- |
| CD105 | Dianova | DLN - 07243 | 1:100 |
| CD73 | Abcam | 7G2 ab54217 | 1:250 |
| **Secondary antibody** | | | |
| **DyLight 488 Donkey Anti-Mouse IgG** | Dianova | 91518 | 1:400 |
